# The Role of DNA Repair in Immunological Diversity: From Molecular Mechanisms to Clinical Ramifications

**DOI:** 10.3389/fimmu.2022.834889

**Published:** 2022-04-01

**Authors:** Peter Gullickson, Yunwen W. Xu, Laura J. Niedernhofer, Elizabeth L. Thompson, Matthew J. Yousefzadeh

**Affiliations:** Department of Biochemistry, Institute on the Biology of Aging and Metabolism, Molecular Biology and Biophysics, University of Minnesota, Minneapolis, MN, United States

**Keywords:** immunological diversity, immunodeficiency, antibodies, DNA damage, DNA repair

## Abstract

An effective humoral immune response necessitates the generation of diverse and high-affinity antibodies to neutralize pathogens and their products. To generate this assorted immune repertoire, DNA damage is introduced at specific regions of the genome. Purposeful genotoxic insults are needed for the successful completion of multiple immunological diversity processes: V(D)J recombination, class-switch recombination, and somatic hypermutation. These three processes, in concert, yield a broad but highly specific immune response. This review highlights the importance of DNA repair mechanisms involved in each of these processes and the catastrophic diseases that arise from DNA repair deficiencies impacting immune system function. These DNA repair disorders underline not only the importance of maintaining genomic integrity for preventing disease but also for robust adaptive immunity.

## Introduction

**Figure 1:**
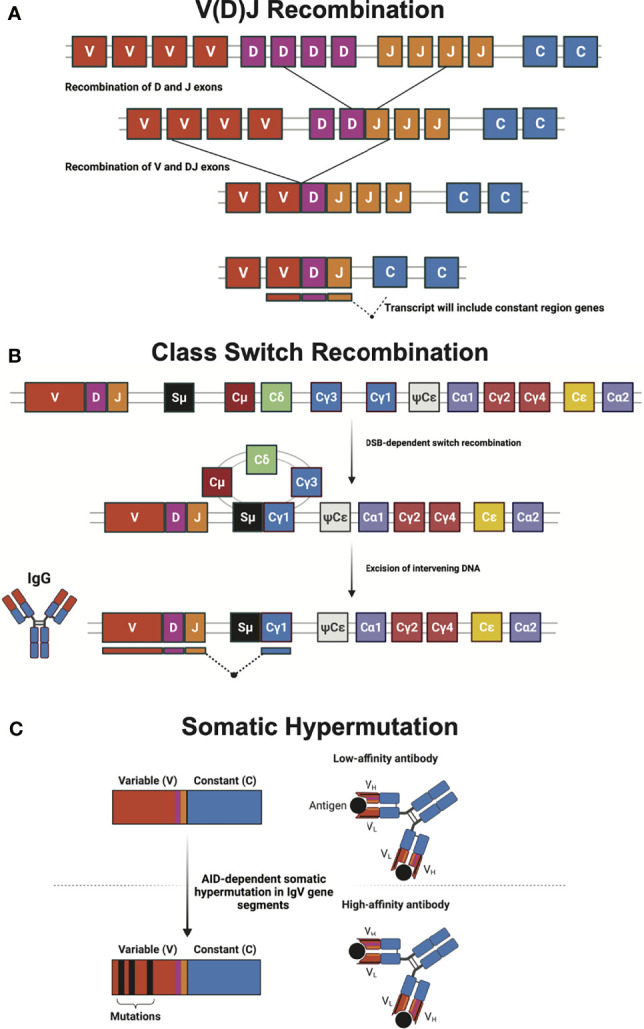
Mechanisms of generating diversity in adaptive immunity. **(A)** V(D)J recombination relies upon RAG-mediated recombination for the rearrangement of immunoglobulin and T cell receptor variable (V), diversity (D), and joining (J) gene segments during lymphocyte development. Many enzymes involved in non-homologous end joining (NHEJ) and other DNA repair mechanisms are required to correct the programmed DNA double-strand breaks (DSB) that initiate gene segment rearrangement. **(B)** Class-switch recombination (CSR) of the immunoglobulin heavy chain locus swaps antibody isotype *via* recombination of different constant (C) regions. CSR requires activation-induced cytidine deaminase (AID) to initiate a DNA DSB break at the switch (S) region, which is subsequently repaired by classical and alternative NHEJ. The schematic shows a CSR event that leads to the production of IgG antibody isotype. **(C)** Somatic hypermutation (SHM) utilizes AID-dependent programmed mutations in the variable region of antibody gene segments to create a large number of antibodies with goal of creating greater affinity for antigen. Antibody heavy (V_H_) and light (V_L_) chains, as well as antigen (black circle) are illustrated. Figure created with BioRender.com.

A functional immune system is defined by a diverse repertoire of cells, surface receptors, and antibodies needed to effectively respond to pathogenic challenges (1). Endogenous DNA damage is a potent driver of disease and aging (2), can trigger innate immune responses, and drive loss of cells *via* apoptosis, necrosis, and senescence (3–5). However, deliberate DNA damage is necessary for vertebrates to respond to the limitless variability of pathogen-related antigens (6, 7). Programmed DNA double-strand breaks (DSB) that occur in B and T cell receptor genes are necessary for lymphocyte development and maturation (6, 8, 9). These programmed DNA breaks occur at specific sites and serve as critical intermediates for rearrangements required for V(D)J recombination (**Figure 1**) (9). Through this process, the nearly 10^12^ B and T cells in an individual express millions of unique combinations of antibody and T-cell receptor genes (10). Immune repertoires of any two individuals may overlap by only a fraction of a percent even though these repertoires are formed by Variable, Diversity, and Joining gene segments that are shared by all humans (11, 12). The diversity between two individuals at the immunoglobulin loci is greater than their germline diversity.

Antibodies (immunoglobulins) directly neutralize pathogens and their gene products (13). In addition, antibodies recruit cellular effectors of immunity to eliminate pathogens and tumor cells. During development, variable regions of the immunoglobulin (*Ig*) locus undergo V(D)J recombination of both the heavy (*IGH*) and light chains (*IGL*) to generate 10^11^ to 10^14^ novel combinations of genetic material (13–15). Upon stimulation, further diversifications of *Ig* genes can be induced by Class Switch Recombination (CSR) and Somatic Hypermutation (SHM) (**Figure 1**). Antibody effector function is governed by its antibody class or isotype. In response to antigen stimulation and costimulatory signaling, programmed DNA damage in the constant region of the *IGH* locus of mature B cells initiates CSR, causing cells to undergo antibody class switching (13, 16). This allows antigen-activated B cells, which are initially IgM^+^ or IgD^+^, to change heavy chain constant domains and express one of the other isotypes encoded downstream in the locus, thus altering antibody function and tissue distribution (6).

Germinal center B cells undergo affinity maturation in lymphoid tissue germinal centers to generate high-affinity antibodies that enable a more effective humoral immune response (17, 18). This process relies upon SHM to generate single point mutations in the *IGH* and *IGL* loci (19, 20). While CSR acts on the constant region of the *IGH* and *IGL* loci, SHM is directed at the variable region. CSR and SHM act in concert to create high-affinity immune responses to each pathogen encountered. VDJ, CSR, and SHM are absolutely dependent on intentional but tightly regulated induction of DNA damage at discrete areas of the genome (20). Multiple components of the DNA repair machinery: sensors, binding proteins, kinases, helicases, recombinases, nucleases, polymerases, and ligases are required for the resolution of the programmed DNA damage that occurs in each of these processes (19, 21). This review highlights the pathophysiological consequences caused by mutations in genes encoding these DNA repair enzymes required for immune diversification.

## V(D)J Recombination

V(D)J recombination is the process that assembles the variable domain of immunoglobulin and T-Cell Receptor (TCR) genes *via* DNA rearrangements (22). V(D)J recombination increases the sequence heterogeneity of a defined gene fragment during the early stages of lymphocyte development. It shapes the immune system repertoire by forming T-cell receptors and immunoglobulins in immature B cells. V(D)J recombination involves multiple DNA repair proteins, including DNA-PKcs, Ku70, Ku80, XRCC4, DNA Ligase IV, and the Cernunnos-XLF protein, all required for non-homologous end-joining of DSBs. Initiation of V(D)J recombination requires lymphoid-specific DNA recombinases RAG1 and RAG2, which recognize recombination signal sequences that flank all V, D, and J gene units and as a complex introduce site-specific DSBs (23–26). MRE11, RAD50, and EXO1 repair proteins are then needed to join the broken DNA ends and resolve the DSB. Mutations in DNA repair factors that participate in V(D)J recombination can severely impact immune function. Mutations in the above genes encoding the above DNA modifying proteins cause varied effects on T and B cell immune cell repertoires. Immunological diseases that arise from DNA repair defects impacting V(D)J recombination are discussed below.

### Severe Combined Immunodeficiency

Severe combined immunodeficiency (SCID) is a rare genetic disorder characterized by impaired development of the immune system and absence of T and B lymphocytes. Mutations in human DNA repair genes *RAG1*, *RAG2*, *DCLRE1C, PRKDC, NHEJ1*, and *LIG4* cause SCID (27). These genes all encode proteins that incise (RAG1-RAG2 complex), excise (*DCLRE1C* Artemis protein) or participate in NHEJ DSB repair (*PRKDC*/DNA-PKcs, *NHEJ1*/XLF4, and LIG4). Loss of function mutations in *RAG1/2, PRKDC*, *KU70*, or *KU80* preclude T and B cell development, leading to SCID (26, 28) (**Table 1**).

**Table 1:** DNA repair deficiency-induced immunological disorders.

Pathway	Disease	Genes	Description	Refs
V(D)J Recombination	Severe Combined Immunodeficiency (SCID)	*RAG1*, *RAG2*, *DCLRE1C, PRKDC, NHEJ1, LIG4*	SCID patients have T and B lymphocyte deficiency. At least 4 diseases can be distinguished by clinical phenotypes and the gene affected.	(18)
V(D)J Recombination	SCID with ARTEMIS deficiency	*DCLRE1C*	Subclinical immunodeficiency: reduction of naïve T cells with increased terminally differentiated T cells due to a reduction in T-cell proliferation. Some patients have reduced B-cell numbers.	(29)
			Hypomorphic mutations in *DCLRE1C* can cause atypical SCID, Omenn syndrome, Hyper IgM syndrome, or inflammatory bowel disease.	
V(D)J Recombination	SCID with Ligase IV deficiency	*LIG4*	Microcephaly and neurodevelopmental delay.	(30)
			T- and B-lymphocytopenia and varying degrees of hypogammaglobulinaemia often associated with high IgM due to defective CSR. Some patients present with features of Omenn’s syndrome and autoimmunity.	
V(D)J Recombination	SCID with Cernunnos-XLF deficiency	*XLF*	T and B-cell lymphopenia, growth retardation, microcephaly, and increased sensitivity to ionizing radiation.	(31)
V(D)J Recombination	SCID with DNA-PKcs deficiency	*PRKDC*	Radiosensitive, growth retardation, microcephaly, and immunodeficiency due to profound T and B cell lymphopenia.	(32, 33)
V(D)J Recombination	Ataxia Telangiectasia (A-T)	*ATM*	Progressive cerebellar degeneration leading to ataxia, telangiectasia*, immunoglobulin deficiency (IgA), lymphopenia (T cells), recurrent sinopulmonary infections, radiation sensitivity, premature aging, and a predisposition to cancer, especially lymphomas.	(34)
			Other abnormalities include poor growth, gonadal atrophy, delayed puberty, and insulin resistance, ataxia: abnormal control of eye movement and postural instability.	
			Telangiectasia: abnormal, tortuous blood vessels	
			(*telangiectasia not present in all A-T patients)	
V(D)J Recombination and, CSR	Ataxia Telangiectasia-like disorder (ATLD)	*MRE11*	Lack of specific functional antibodies causing minimal immunodeficiency, ataxia, and dysarthria.	(35)
V(D)J Recombination	Nijmegen breakage syndrome	*NBN*	Progressive microcephaly presenting *in utero*, dysmorphic facial features, mild growth retardation, mild-to-moderate intellectual disability, and, in females, hypergonadotropic hypogonadism.	(36, 37)
			Immunodeficiency (decreased T cells and reduced IgG/IgA) and a high incidence of pediatric malignancies, mostly lymphomas and leukemias.	
CSR and NHEJ	RIDDLE syndrome	*RNF168*	Radiosensitivity, Immunodeficiency, Dysmorphic features, and Learning difficulties, increased serum IgM and reduced IgG levels.	(38, 39)
CSR, SHM, BER	Hyper IgM Syndrome Type 5	*UNG*	Elevated serum IgM with low IgG and IgA, increased susceptibility to bacterial infections and lymphoid hyperplasia.	(40)
CSR and SHM	Hyper IgM Syndrome Type 2	*AICDA*	Elevated serum IgM levels, low IgG, low IgA. lymphoid hyperplasia, and recurrent infections.	(41)
CSR and MMR	*PMS2* or *MSH6* deficiency	*PMS2*	Elevated serum IgM and low IgG and IgA, recurrent infections, cafe-au-lait spots. Associated with Lynch Syndrome and colorectal and endometrial cancer.	(42, 43)

Artemis deficiency, caused by null mutations in *DCLRE1C*, also causes SCID. Artemis is an exonuclease essential for the repair of DSBs *via* non-homologous end-joining (NHEJ) and plays a critical role in V(D)J recombination. Artemis mutations create a broad spectrum of phenotypes that range from SCID to antibody deficiency (29, 44, 45). NK cell number and function are unaffected in Artemis-deficient SCID patients. However, these patients commonly have radiation sensitivity consistent with a DSB repair defect (44). The impact of a mutation on NHEJ repair and capacity can vary between individuals with mutations in *DCLRE1C* and do not correlate well with clinical severity (46).

DNA-PKcs is a key component of DNA Protein Kinase complex (DNA-PK), which plays a critical role in NHEJ. Artemis is a substrate for DNA-PKcs kinase activity and phosphorylation is required for its nuclease activity that cleans up broken DNA ends. Artemis binds DNA-PKcs and the Artemis-DNA-PK complex cleaves 5’ and 3’ overhangs of hairpins generated by the RAG complex. Mutations in *PRKDC* can also impair Artemis activation or its ability to bind DNA ends during DSB repair. DNA-PK also has a role in recruiting other NHEJ proteins like XRCC4 and LIG4 to DSBs. As the NHEJ pathway is critical in V(D)J recombination, hypomorphic and null mutations in *PRKDC* lead to dysfunction in the development of T and B cells. *PRKDC* mutations were only discovered relatively recently in a SCID patient that exhibited symptoms similar to patients with *RAG* or *DCLRE1C* mutations (32). The patient was practically devoid of B and T cells while NK cell numbers were normal. The patient did not display signs of microcephaly or intellectual disability observed in other DNA repair disorders impacting the immune system (32).

DNA ligase IV syndrome, which has features of SCID, is caused by a *LIG4* deficiency. This rare autosomal recessive disorder is characterized by microcephaly, abnormal facial features, sensitivity to ionizing radiation, and SCID (30). Only 30 patients with Ligase IV syndrome have been described, and while they all are sensitive to ionizing radiation (47), they exhibit a broad spectrum of clinical features. Patients typically exhibit low T and B cell numbers and low serum Ig levels, resulting in immunodeficiency (30).

### Ataxia Telangiectasia

Ataxia Telangiectasia (A-T) is a genetic neurodegenerative disorder that is characterized by progressively cerebellar atrophy with impaired coordination of voluntary movements (ataxia), the development of reddish lesions of the skin and mucous membranes due to dilation of blood vessels (telangiectasia), and immune dysfunction (cellular and humoral immunodeficiency resulting in increased susceptibility to infections, cancer and malignancies, in particular lymphoid malignancies) (34). A-T is caused by mutations in the AT-mutated *(ATM)* gene, the gene product of which is a key component of the DNA damage response. Mutations in *ATM* cause aberrant V(D)J recombination and apoptosis during lymphocyte development, resulting in patients having immunoglobulin deficiencies and lymphopenia (48–50). A-T patients with inactivating mutations in *ATM* sporadically have T cell prolymphocytic leukemia (T-PLL), B cell chronic lymphocytic leukemia (B-CLL), and mantle cell lymphoma (MCL) (51). CSR deficiency is also characteristic of A-T, resulting in high serum IgM levels, with low IgA and IgG levels (35).

### Nijmegen Breakage Syndrome

Nijmegen breakage syndrome (NBS) is a rare autosomal recessive syndrome of chromosomal instability mainly characterized by microcephaly at birth, SCID, and a predisposition to malignancies. It is caused by mutations in *NBN*, which encodes NBS1 (36, 52). NBS1 forms a multimeric complex with MRE11 and RAD50 nuclease (MRN complex) *via* its C-terminus. The function of NBS1 is to recruit and retain the complex at sites of DNA damage by directly binding to histone H2AX, a histone phosphorylated by PI3-kinase family members such as ATM, in response to DNA damage. The MRN complex facilitates the rejoining of DBSs predominantly by homologous recombination repair rather than NHEJ (52, 53). NBS patients have variability in immunodeficiency, as the number of CD8^+^ T cells could be normal, elevated, or considerably reduced, with decreased CD4^+^ T cell counts. However, universally there is an increase in unresolved recombination-mediated breaks in *IGH* and a compensatory proliferation of mature B cells as absolute B cell numbers are decreased, consistent with a V(D)J recombination defect (36, 37).

## Class Switch Recombination

The ability of the immune system to fight and eliminate a wide array of pathogens is made possible by the production of a variety of antibody isotypes, each with unique effector functions. Naïve B-cells produce only membrane-bound antibodies IgM and IgD. Following infection, naïve B cells are activated and can be induced to undergo CSR (13, 18, 54). CSR occurs in the DNA encoding the constant region of *IGH* (16). Here, deletional recombination occurs between DSBs intentionally introduced at switch (S) regions between *IGH* constant region genes (18) (**Figure 1**).

The process of introducing DSBs begins with activation-induced cytidine deaminase (AID), which demethylates cytosines to uracil at immunoglobulin switch regions (55, 56). Next, uracil-DNA glycosylase (UNG), a component of the base excision repair (BER) pathway, excises the uracils, leaving abasic sites that are further processed to create DNA single strand breaks (SSB) (57, 58). If SSBs occur in both strands of the DNA in close proximity, then a DSB results. DNA mismatch repair (MMR) can also create DSBs following AID-induced demethylation (16). MMR recognizes U:G mismatches and resects single-stranded DNA created by mismatch-induced DNA unwinding. If there is a SSB on the opposite strand in the resected region, then a DSB is introduced. The DSBs at the switch regions are recognized, recombined, and then repaired using primarily NHEJ, similar to VDJ recombination (59). In CSR, alternative end-joining (A-EJ) also plays a role in repairing DSBs (60). In contrast to the classical NHEJ (c-NHEJ), A-EJ is a relatively slower and more error-prone process that relies upon annealing at microhomologies. A-EJ is also considered as a prominent source of genome instability (59). A-EJ is substantially less efficient than NHEJ but enables CSR in c-NHEJ-deficient cells (60). Many factors including, stage of the cell cycle, also influence which repair pathway is utilized (61). Some DNA repair factors have distinct contributions in A-EJ versus c-NHEJ. For example, 5-Hydroxymethylcytosine binding, ES cell-specific-protein (HMCES) is dispensable for c-NHEJ but the significant CSR defect observed in HMCES-deficient primary B cells is due to its downstream role in A-EJ (62). Elevated end-resection, non-productive interchromosomal translocations and inversions were observed during sequence analysis of CSR junctions of kinase-dead DNA-PKcs but not DNA-PKcs-deficient B cells (63). ERCC1-XPF, whose role in CSR is not fully understood, removes non-homologous 3’ overhangs that result from annealing at microhomologies during A-EJ (64).

While most CSR-related diseases (discussed below) result from non-functional CSR proteins, the initiation of AID-induced damage outside of the *IGH* locus can lead to translocations and B cell lymphomas (65–68). Beyond AID’s role in CSR, it also participates in a phenomenon called locus suicide recombination (LSR) which abolishes B cell function (69, 70). In LSR, AID initiates recombination between the most upstream *IGH* switch region (Sμ) and a “switch-like” region near the 3’ regulatory region resulting in the deletion of the *IGH* constant region, rendering the B cell non-viable. Although its regulation is not well understood, the balance between CSR and LSR may play a critical role in B cell fate. These studies illustrate the deleterious aspects of AID-mediated recombination that yield non-productive antibodies and B cell death (69, 71).

### DNA Repair Syndromes Affecting CSR

DNA repair is critical for antibody diversification through CSR, which is evident in the numerous CSR-related diseases caused by mutations in DNA repair proteins (**Table 1**). When CSR is not functioning properly, individuals exhibit immunodeficiency due to an impaired ability of B-cells to switch to IgA, IgG, and/or IgE production. The characteristic phenotype of CSR-related diseases is elevated serum IgM levels with low IgA, IgG, and/or IgE levels (6, 13). There is substantial variation in clinical phenotypes both within a disease and between diseases with impaired CSR. For example, a study of patients with MSH6 deficiency found that one patient had elevated IgM levels and reduced IgG, four had elevated IgM and normal IgG, two had normal IgM and reduced IgG, and one had normal IgM and normal IgG (72).

Although AID is not technically a DNA repair protein, its intentional introductions of DNA damage are crucial for the initiation of CSR. Mutations in *AICDA*, the gene that encodes AID, cause hyper-IgM syndrome (HIGM) type 2 (41). Patients with HIGM type 2 typically present with elevated serum IgM levels with low IgA and IgG levels (73, 74). Following the replacement of cytosine DNA bases in switch regions with uracil by AID, BER and MMR proteins play critical roles in producing DSBs. Mutations in *UNG*, coding for the BER protein UNG, result in the HIGM Type 5 (**Table 1**). Like HIGM type 2, this syndrome is characterized by high serum IgM levels, low IgG levels, and low IgA levels (40, 42). Additionally, patients with PMS2- and MSH6-driven MMR deficiency exhibit defective CSR, which is also the case in MLH1 and MSH5-deficient mice (**Table 1**) (43, 72, 75, 76).

Defects in DSB recognition and signaling proteins can cause CSR-related immunodeficiency. The MRN complex (MRE11-RAD50-NBS1) recognizes DSBs and activates ATM, the key transducer of signaling in response to DSBs. Ataxia Telangiectasia (A-T), Ataxia Telangiectasia-Like Disorder (A-TLD), and NBS, caused by mutations in *ATM, MRE11*, and *NBS1*, respectively, all lead to CSR defects (34, 35). NBS patients have a defect in CSR as well as VDJ recombination. A-T and A-TLD share many clinical phenotypes such as ataxia, dysarthria, and abnormal eye movements. However, A-T and NBS result in more similar immunodeficiency phenotypes than A-T and A-TLD. Patients with A-T and NBS often exhibit elevated serum IgM levels, low IgA levels, and low IgG levels (**Table 1**) (77–79). In contrast, A-TLD patients exhibit very mild immunodeficiency, with reductions in some specific antibody isotypes observed (35, 80). RNF168 is another protein involved in signaling and repair protein recruitment following recognition of DSBs (38). RIDDLE syndrome is caused by *RNF168* mutations and is characterized by defective CSR resulting in low serum IgG levels (39). Mutations affecting critical NHEJ proteins often cause CSR-related immunodeficiency. Low or absent serum IgA and IgG levels are common in Cernunnos-XLF- and DNA-PKcs-deficient patients (81, 82). In addition, DNA Ligase IV deficiency often results in low serum IgG levels (30) (**Table 1**).

## Somatic Hypermutation

SHM is another example of intentional DNA damage being induced to enable antibody diversification in germinal center B cells. SHM introduces point mutations in the *Ig* locus primarily in the antibody variable (V) region that codes for the antigen-binding site of immunoglobulin heavy and light chains (**Figure 1**). This allows for the production and selection of B cells with high-affinity antibodies (17, 83, 84). The mutation frequency in SHM is a million-fold higher than the basal genome mutation rate. How B cells restrict SHM to the V region while maintaining genome-wide integrity is not well understood. AID initiates antibody affinity maturation through SHM, analogous to initiating CSR. Centroblast B cells in the germinal centers of lymphoid organs express large amounts of AID to initiate SHM (85). Numerous point mutations occur at both the site of uracil incorporation and proximal nucleotides through three predominant mechanisms: replication, BER, and MMR. Uracil incorporated by AID can persist into the S phase during which DNA replication can result in C to T (or G to A) transition mutations (86). However, replication accounts for less than half of all the mutations incorporated during SHM (83). Error-prone non-canonical BER and MMR can combine to diversify mutations introduced during SHM (87, 88). Similar to CSR, SHM-associated uracils are excised by UNG creating an abasic site during BER. Abasic sites are then bypassed by an error-prone translesion synthesis (TLS) DNA polymerase, like Rev1, which can introduce C:G transversion (88, 89). Alternatively, a non-canonical MMR pathway can recognize and repair AID-induced U:G mispairs. This pathway utilizes the error-prone TLS DNA polymerase η, which primarily creates mutations at A:T base pairs (88, 90, 91). Inactivating mutations in *AID* can result in HIGM type 2 and *UNG* mutations can result in HIGM type 5 (41, 57, 92). In both conditions, the patients have defects in CSR and SHM and are susceptible to infections (**Table 1**).

## Conclusion

While genotoxic injury is looked upon as unfavorable, it is quite beneficial for certain processes like meiotic recombination and immunological diversity. Deliberate induction and repair of DNA damage serve as a catalyst to expand our immune repertoire. V(D)J and class-switch recombination yield unique antibody combinations and establish effector function (6). Both pathways incorporate many components of the DNA damage response, recombinases, and enzymes from NHEJ repair pathway in addition to other components of the DNA repair machinery, including helicases, nucleases, polymerases, and ligases. Lastly, intentional *de novo* mutations in the variable region of immunoglobulin genes by SHM create high-affinity antibodies. While DNA repair-deficient murine models have been used to explore disease mechanisms and driver events in tumorigenesis, samples from DNA repair disorder patients have provided great insight into the functional consequences of impaired DNA damage on diversification and development of the adaptive immune system. Future exploration to investigate immune perturbations in other monogenic diseases of DNA repair may provide insight into other DNA repair mechanisms that contribute to immune responses.

## Author Contributions

PG, YWX, and MJY prepared the figure and table. PG, YWX, MJY, ELT, and LJN wrote the manuscript. The order of co-first authorship was chosen alphabetically. All authors contributed to the article and approved the submitted version.

## Funding

This work is supported by funds from the NIH (U01 ES029603, R01 AG063543, P01 AG062413, and U19 AG056278) to LJN. ELT is supported by funds from the NIH T32 AG029796. MJY is supported by the Irene Diamond Fund/American Federation for Aging Research Postdoctoral Transition Award.

## Conflict of Interest

LJN is a co-founder of NRTK Biosciences, a start-up biotechnology company developing senolytic drugs.

The remaining authors declare that the research was conducted in the absence of any commercial or financial relationships that could be construed as a potential conflict of interest.

## Publisher’s Note

All claims expressed in this article are solely those of the authors and do not necessarily represent those of their affiliated organizations, or those of the publisher, the editors and the reviewers. Any product that may be evaluated in this article, or claim that may be made by its manufacturer, is not guaranteed or endorsed by the publisher.
